# Expression of *EEF1A1* Is Associated with Prognosis of Patients with Colon Adenocarcinoma

**DOI:** 10.3390/jcm8111903

**Published:** 2019-11-07

**Authors:** Eun kyo Joung, Jiyoung Kim, Nara Yoon, Lee-so Maeng, Ji Hoon Kim, Sungsoo Park, Keunsoo Kang, Jeong Seon Kim, Young-Ho Ahn, Yoon Ho Ko, Jae Ho Byun, Ji Hyung Hong

**Affiliations:** 1 Department of Internal Medicine, College of Medicine, The Catholic University of Korea, Seoul 06591, Korea; dmsrysla0410@gmail.com; 2 Department of Pathology, Incheon St. Mary’s Hospital, College of Medicine, The Catholic University of Korea, Seoul 06591, Korea; jypath@catholic.ac.kr (J.K.); narayoon@catholic.ac.kr (N.Y.); mls1004@catholic.ac.kr (L.-s.M.); 3 Department of General Surgery, Incheon St. Mary’s Hospital, College of Medicine, The Catholic University of Korea, Seoul 06591, Korea; samryong@catholic.ac.kr; 4 Deargen Inc., Daejeon 34051, Korea; sspark@deepimagine.com; 5 Department of Microbiology, College of Natural Sciences, Dankook University, Cheonan 31116, Korea; kangk1204@gmail.com; 6 Department of Molecular Medicine and Tissue Injury Defense Research Center, College of Medicine, Ewha Womans University, Seoul 03760, Korea; jeongseonkim821@gmail.com (J.S.K.); yahn@ewha.ac.kr (Y.-H.A.); 7 Division of Oncology, Department of Internal Medicine, Eunpyeong St. Mary’s Hospital, The Catholic University of Korea, Seoul 03312, Korea; koyoonho@catholic.ac.kr; 8 Cancer Research Institute, College of Medicine, The Catholic University of Korea, Seoul 06591, Korea; 9 Division of Oncology, Department of Internal Medicine, Incheon St. Mary’s Hospital, College of Medicine, The Catholic University of Korea, Seoul 06591, Korea

**Keywords:** cancer biomarker, colon adenocarcinoma, elongation factor-1 alpha 1, prognosis, translation factor

## Abstract

Background: The prognostic role of the translational factor, elongation factor-1 alpha 1 (EEF1A1), in colon cancer is unclear. Objectives: The present study aimed to investigate the expression of EEF1A in tissues obtained from patients with stage II and III colon cancer and analyze its association with patient prognosis. Methods: A total of 281 patients with colon cancer who underwent curative resection were analyzed according to EEF1A1 expression. Results: The five-year overall survival in the high-EEF1A1 group was 87.7%, whereas it was 65.6% in the low-EEF1A1 expression group (hazard ratio (HR) 2.47, 95% confidence interval (CI) 1.38–4.44, *p* = 0.002). The five-year disease-free survival of patients with high EEF1A1 expression was 82.5%, which was longer than the rate of 55.4% observed for patients with low EEF1A1 expression (HR 2.94, 95% CI 1.72–5.04, *p* < 0.001). Univariate Cox regression analysis indicated that age, preoperative carcinoembryonic antigen level, adjuvant treatment, total number of metastatic lymph nodes, and EEF1A1 expression level were significant prognostic factors for death. In multivariate analysis, expression of EEF1A1 was an independent prognostic factor associated with death (HR 3.01, 95% CI 1.636–5.543, *p* < 0.001). EEF1A1 expression was also an independent prognostic factor for disease-free survival in multivariate analysis (HR 2.54, 95% CI 1.459–4.434, *p* < 0.001). Conclusions: Our study demonstrated that high expression of EEF1A1 has a favorable prognostic effect on patients with colon adenocarcinoma.

## 1. Introduction

Colon cancer is the second leading cause of cancer-related death worldwide, with an incidence of 9.2% [1]. Although curative surgical resection of primary lesions and post-operative adjuvant treatment contribute to the prevention of early recurrence in patients with high-risk stage II and III colon cancer, the recurrence rate after curative surgery remains at 20%–30% [2]. To prolong disease-free survival (DFS) after curative surgery and avoid unnecessary over-treatment of patients, more accurate prognostic tools are required. However, a few clinical prognostic factors, such as the presence of obstructive symptoms, number of retrieved regional lymph nodes, pathologic tumor and nodal stage, and features associated with micrometastatic potential including vascular, lymphatic, and perineural invasion, have been used to select patients that may benefit from adjuvant therapies [3]. Although other molecular signatures, such as microsatellite status, BRAF (v-Raf murine sarcoma viral oncogene homolog B) and KRAS (V-Ki-ras2 Kirsten rat sarcoma viral oncogene homolog) mutations, molecular signatures, and immune infiltration, have been suggested as potential prognosticators, more sophisticated prognostic biomarkers need to be identified [1,3].

To identify accurate and definitive molecular biomarkers for human malignancies, we previously developed a neural network-based model, Wx, which is optimized to select gene sets based on the discriminative index between combination pairs of K class [4]. In our previous report, we applied our Wx model to a pan-cancer cohort from The Cancer Genome Atlas (TCGA) RNA-Seq dataset, consisting of 12 different types of cancer and normal samples, and obtained 14 genes distinguishing cancer tissues from normal tissues with high accuracy. Tissue elongation factor 1 alpha 1 (*EEF1A1*) was one of the 14 biomarker genes extracted using the Wx model. Interestingly, it was also identified by Martinez-Ledesma et al. using the same discriminative concept to compare colon adenocarcinoma to normal tissues [5].

EEF1A1 is a ubiquitous protein that functions in peptide elongation during mRNA translation and is the second-most abundant protein after actin in eukaryotic proteomes [6,7]. As a translational factor, EEF1A delivers the aminoacylated-tRNA to the ribosome, as a ternary complex comprising EEF1A–GTP (Guanosine-5’-triphosphate)–aminoacylated-tRNA, to decode mRNA. There are two isoforms of *EEF1A*, namely *EEF1A1* and *EEF1A2*, which were mapped to chromosomes 6q14 and 2q13.3, respectively [8]. *EEF1A1* and *EEF1A2* share more than 95% DNA and protein identity [9]. EEF1A1 is expressed in most cells, whereas EEF1A2 is present only in the brain, heart, and skeletal muscle [10]. Although the functional significance of their tissue-specific expression patterns is unknown, they are thought to have the same enzymatic function in protein translation. Several studies have revealed that EEF1A is not only a translation factor but also involved in many non-canonical functions including oncogenesis, protein degradation, pro-apoptotic or anti-apoptotic activity, and cytoskeleton modulation [10,11,12,13]. Notably, many studies have shown that *EEF1A* is a prognostic factor for several solid tumors such as ovary [11,14], breast [15,16,17], lung [18], pancreas [19,20], stomach [21,22], prostate [23], and liver cancers [24,25]. Based on the previous results from the Wx neural network-based feature selection algorithm and those reporting the role of EEF1A1 in human solid cancer, we hypothesized that EEF1A1 is related to the prognosis of patients with colon adenocarcinoma. In this study, we investigated the expression of EEF1A in tissues from patients with stage II and III colon cancer and analyzed its association with patient prognosis.

## 2. Methods

### 2.1. Identification of Prognostic Biomarker Genes Using the Wx Algorithm with TCGA Database

Genes distinguishing cancer from normal samples were identified by applying the Wx algorithm to a pan-cancer cohort containing 6210 samples with 12 different types of cancer, using mRNA-Seq data from TCGA. In this study, we re-analyzed the mRNA-Seq data of 327 colon adenocarcinomas (287 cancer and 40 normal samples) to identify biomarker candidate genes using the Wx algorithm. The Wx algorithm ranks genes based on the discriminative index score, which reflects the classification power of distinction between groups (e.g., cancer vs. normal). The detailed method has been described previously [4].

### 2.2. Patients and Tissue Samples

Medical records of patients with colon cancer who have undergone curative surgery at Incheon St. Mary’s hospital between 2010 and 2013 were reviewed. Their tissue microarrays (TMAs) for immunohistochemistry were obtained. If available, fresh-frozen tumor tissues and paired normal adjacent tissues from patients were used for RNA extraction. Demographic and clinicopathological data for these patients were reviewed retrospectively from the medical records. Variable factors including age, sex, sidedness of colon cancer, pathologic staging, histology, and lymphatic, venous, and perineural invasion were analyzed, and tumors were staged according to the pathological tumor/node/metastasis (pTNM) classification (8th edition) of the Union for International Cancer Control. The study was approved by the Institutional Review Board of Incheon St. Mary’s hospital, the Catholic University of Korea (OC15TISI0050). Informed consent was waived considering the retrospective study design.

### 2.3. Quantitative Reverse Transcription PCR (qRT-PCR)

Total RNA was isolated from tumors and adjacent normal tissues of patients with colon cancer using the WelPrep^TM^ Total RNA Isolation Reagent (Welgene, Daegu, Korea) and gentleMACS Dissociator (Miltenyi Biotec, Bergisch Gladbach, Germany), according to the manufacturer’s protocols. To analyze mRNA levels, qRT-PCR assays were performed using a BioFACT^TM^ A-Star Real-time PCR Kit including SFCgreen® I (BioFACT, Daejeon, Korea) after reverse transcription with ELPIS RT Prime Kit (Elpis-Biotech, Daejeon, Korea). mRNA levels were normalized to those of ribosomal protein L32 (*RPL32*).

### 2.4. Tissue Microarray Construction and Immunohistochemistry

Formalin-fixed paraffin-embedded colon cancer samples from patients who underwent surgery were included in a TMA after careful review by two experienced pathologists. TMAs were prepared with a tissue array and were composed of 69 cores per TMA, with a core diameter of 2 mm. The cores were derived from separate sources and treated as individual cases, and not as duplicates from a single source. Cases that presented as colon cancer had sufficient material for two cores, whereas normal tissues could be used to generate one core. A set comprising 14 slides was stained by immunohistochemistry. The antibodies used for the study included a mouse monoclonal antibody against EEF1A1 (sc-21758) from Santa Cruz Biotechnology, Inc. (Dallas, TX, USA), which was used according to the manufacturer’s instructions at a dilution of 1:100 after optimization. Immunohistochemical staining was carried out with the Ventana BenchMark ULTRA (Tucson, AZ, USA) using the ultraView Universal DAB detection kit. This included standard de-paraffinization, blocking of endogenous peroxidase and biotin, incubation with primary and secondary antibodies, and development with DAB (di-aminobenzidine). The slides were counterstained with hematoxylin and eosin (H&E).

### 2.5. Evaluation of Immunohistochemical Staining of EEF1A1

**Figure 1:**
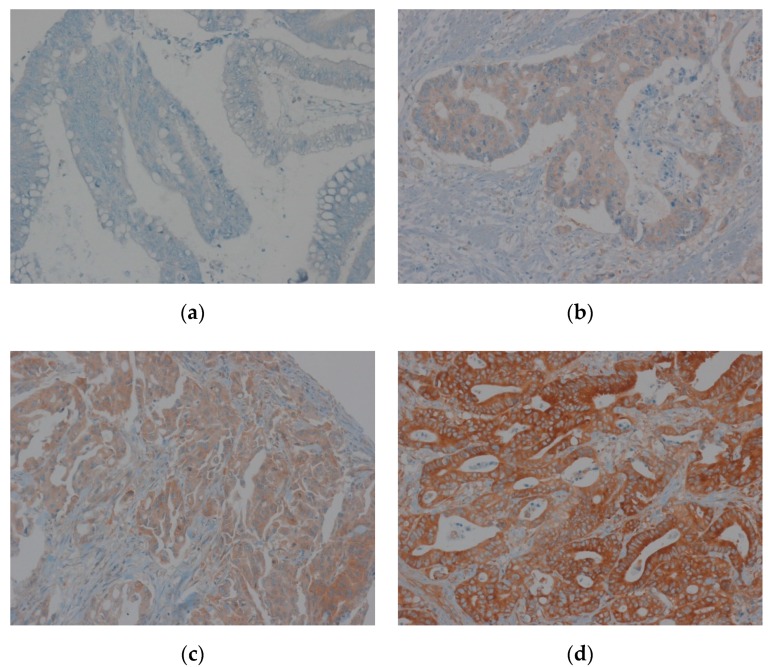
Immunohistochemical staining of elongation factor-1 alpha 1 (EEF1A1) in colon adenocarcinoma tissue. (**a**) Staining intensity 0; (**b**) staining intensity 1; (**c**) staining intensity 2; (**d**) staining intensity 3.

Immunohistochemical staining for EEF1A1 revealed its cytoplasmic expression in most tumor cells. Staining intensity was graded from 0 to 3 (0 = no expression, 1 = weak, 2 = moderate, and 3 = strong). The extent was graded from 0 to 2 (0 = 0%, 1 = 1%–50%, 2 = 51%–100% of cells) (Figure 1). The intensity and extent scores were added to obtain a composite score. Patients with composite scores of 0–2 were classified into the low-EEF1A1 expression group and those with scores of 3–6 were included in the high-expression group. The results were analyzed by two pathologists who were blinded to the clinical data.

### 2.6. Statistical Analysis

Categorical values were presented as means and percentages, and non-categorical values were presented as median values and ranges. The Chi-squared test and one-way analysis of variance were performed to compare differences among means and medians, respectively. The Kaplan–Meier method was used to calculate survival rates. DFS was defined as the period from the date of curative operation of colon cancer to the date of recurrence based on medical records. Overall survival (OS) was defined as the period from the date of curative operation of colon cancer to the date of death due to any cause. Right colon cancer was defined as cancer originating from the ascending colon to the splenic flexure and left colon cancer was defined as originating from the descending colon to the sigmoid colon. A *p*-value < 0.05 was considered to indicate statistical significance. Differences in survival rates across each patient group were examined by the log-rank test. A Cox proportional-hazards model was used for univariate and multivariate analyses. All analyses were performed with SPSS for windows (version 25.0, SPSS, Inc., Chicago, IL, USA).

## 3. Results

### 3.1. Baseline Characteristics of the Study Population

A total of 281 patients with colon cancer who underwent curative resection as the primary treatment were retrospectively analyzed. Of them, 132 patients (47.0%) had stage II disease, whereas the remaining 149 (53.0%) had stage III lesions. Among patients with stage II, 89 (67.4%) were administered post-operative adjuvant treatment. The median pre-operative carcinoembryonic antigen (CEA) value was 3.34 ng/mL (range: 0.08–1926.6 ng/mL). The *KRAS* status was identified in only 143 patients and *KRAS* mutations were observed in 52 (36.4%) tumor tissues. The detailed demographic features of these patients are summarized in Table 1.

### 3.2. mRNA Expression of EEF1A1 in Human Colon Cancer Tissues Compared to That in Normal Adjacent Tissues

**Figure 2:**
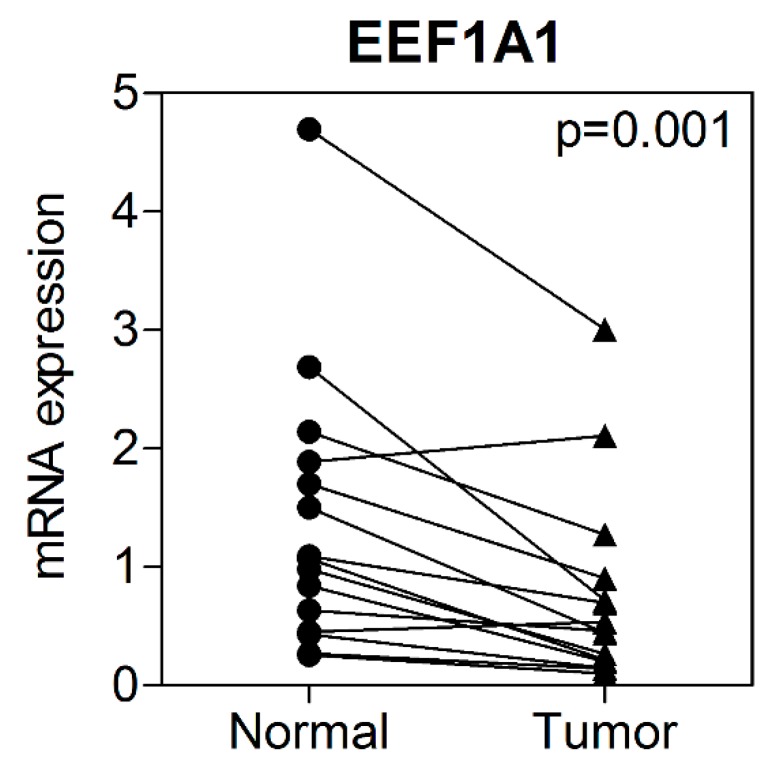
Expression of EEF1A1 in colon cancer tissue and normal adjacent tissue.

Of the total study population, *EEF1A1* mRNA expression levels were investigated in 15 patients from whom fresh tumor and adjacent normal tissue could be harvested. *EEF1A1* mRNA levels were significantly reduced in the tumor tissues compared to those in the normal adjacent tissue (Figure 2).

### 3.3. Correlation between EEF1A1 Expression and Clinicopathological Characteristics

EEF1A1 immunostaining was typically negative or very weakly positive in the perinuclear cytoplasmic area of the normal colonic mucosa. In the tissue with positive EEF1A1 immunostaining, diffuse cytoplasmic staining was observed in tumor cells. The intensity of EEF1A1 was as follows: intensity 0:19 patients (6.8%), 1:86 (30.6%), 2:139 (49.5%), and 3:37 (13.2%). According to composite scores, 42 (14.9%) patients were classified as having low EEF1A1 expression, whereas 239 patients (85.1%) were classified as having high expression (Table S1). Stage, age, sex, harvested lymph node during surgery, and the proportion of patients administered post-operative adjuvant treatment did not differ between patients with low and high expression. Intestinal obstruction or perforation, due to primary colon cancer, was more frequently observed in patients with low EEF1A1 expression than in those with high expression. Patients with low EEF1A1 expression tended to have a larger proportion of right-sided tumors than those with high expression, although this result was not significant. KRAS mutations were not associated with EEF1A1 expression (*p* = 0.399). Other patient clinicopathological characteristics, according to EEF1A1 expression, are listed in Table 1.

### 3.4. Survival Outcomes According to EEF1A1 Expression

**Figure 3:**
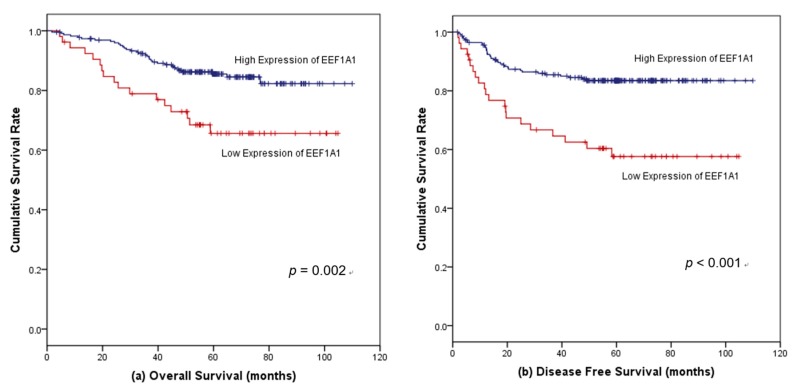
Kaplan–Meier survival curves according to EEF1A1 protein expression in all colon cancer patients. (**a**) High EEF1A1 expression was significantly associated with favorable overall survival (OS) and (**b**) disease-free survival (DFS).

The median follow-up period was 59.9 months (range 1.7–110.0 months). Considering all patients, the five-year OS in the high-EEF1A1 group was 87.7%, whereas it was 65.6% in the low-EEF1A1 expression group (*p* = 0.002, hazard ratio (HR) = 2.47, 95% confidence interval (CI) = 1.38–4.44) (Figure 3a). The five-year DFS of patients with high EEF1A1 expression was 82.5%, which was longer than the rate of 55.4% observed for patients with low EEF1A1 expression (*p* < 0.001, HR = 2.94, 95% CI = 1.72–5.04) (Figure 3b).

**Figure 4:**
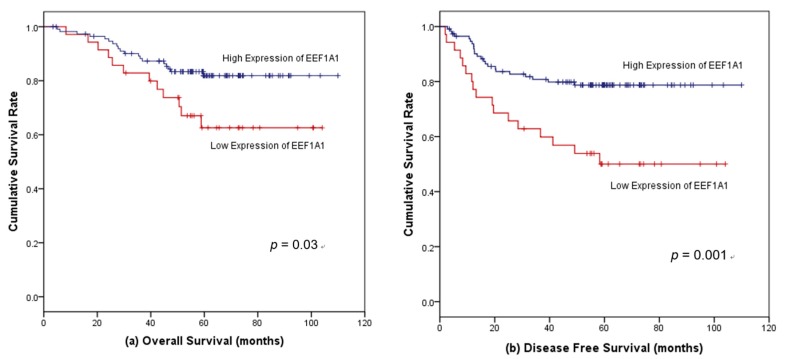
Kaplan–Meier survival curves according to EEF1A1 protein expression in stage III colon cancer patients. (**a**) High EEF1A1 expression was significantly associated with favorable overall survival (OS) and (**b**) disease-free survival (DFS).

For stage II cancer, the 5-year OS of patients with high EEF1A1 expression tended to be longer than that of patients with low EEF1A1 expression (89.0% vs. 71.1%, *p* = 0.051, HR = 2.68, 95% CI = 0.96–7.43). The 5-year DFS of patients with high EEF1A1 was 88.0%, compared to 75.4% for those with low EEF1A1 (*p* = 0.134, HR = 2.30, 95% = CI 0.75–7.07). Among patients with stage II disease who were not administered post-operative adjuvant treatment, those with high EEF1A1 expression had a longer OS (5-year OS rate, 87.7%) than those with low EEF1A1 levels (57.1%) (*p* = 0.026). This survival difference according to EEF1A1 expression was not observed for patients administered adjuvant treatment after stage II disease (*p* = 0.985). For stage III cancer, the 5-year OS was 88.1% for patients with high EEF1A1 expression and 62.6% for those with low EEF1A1 (*p* = 0.030, HR = 2.22, 95% CI = 1.06–4.50) (Figure 4a). Among patients with stage III disease, the 5-year DFS for patients in the high-EEF1A1 group was 78.7%, compared to 50.0% for those with low EEF1A1 (*p* = 0.001, HR = 2.72, 95% = CI 1.45–6. 09) (Figure 4b).

### 3.5. Factors Associated with Survival in Patients with Stages II and III Colon Cancer

Univariate Cox regression analysis indicated that age, pre-operative CEA levels, adjuvant treatment, total number of metastatic lymph nodes, and EEF1A1 expression levels were significant prognostic factors for death. Based on multivariate analysis, the expression of EEF1A1 retained its independence as a prognostic factor associated with death (HR = 3.01, 95% CI = 1.636–5.543, *p* < 0.001) (Table 2). EEF1A1 expression was also an independent prognostic factor for recurrence based on multivariate analysis (HR = 2.54, 95% CI = 1.459–4.434, *p*-value < 0.001). Other significant prognostic markers for DFS included pre-operative CEA level and TNM stage (Table 3). 

## 4. Discussion

*EEF1A1* was a biomarker used to distinguish between cancer and normal tissue in our previous study, using a neural network-based feature selection algorithm. In the present study, we investigated EEF1A1 expression and its prognostic significance for patients with stage II and III colon cancer who underwent curative resection. The results revealed that *EEF1A1* mRNA was decreased in tumor tissue compared to that in normal tissue. Patients with low expression of EEF1A1 showed poorer survival outcomes in terms of both DFS and OS compared to those with higher expression. The expression level of EEF1A1 was found to be an independent prognostic factor for survival outcomes. Among stage II patients not administered adjuvant treatment, a poorer OS outcome was observed for those with low EEF1A1 expression than for those with high EEF1A1 expression. Among patients with stage III colon cancer, those with low EEF1A1 expression had a shorter OS than those with high EEF1A1 expression. We determined the prognostic effect of EEF1A1 on human colon cancer. To the best of our knowledge, this is the first study to investigate the prognostic value of EEF1A1 expression in human colon cancer. 

The physiological role of EEF1A1 in eukaryotic protein synthesis involves recruiting amino-acylated tRNA to the ribosome during the elongation phase of translation. It also plays various non-canonical roles including cytoskeleton modulation, protein degradation, apoptosis, and oncogenesis [12,13]. Particularly, EEF1A1 has been reported to be a pro-apoptotic protein in previous studies. Ruest et al. reported that myotube death is accelerated by the induction of the homologous gene [26], *EEF1A-1/EF-1α.* Borradaile et al. demonstrated that induction of *EEF1A1* is involved in lipotoxic cell death secondary to oxidative stress [27]. Bosutti et al. compared the mRNA levels of *EEF1A1*, *EEF1A2*, and other pro-apoptotic genes (*p66* and *c-MYC*) in skeletal muscles between severely traumatized patients and healthy volunteers [28]; the results showed upregulation of muscle *EEF1A1* and *p66* in the traumatized patient group, which was significantly related to the proteolysis rate. Thus, pro-apoptotic function is a possible mechanism underlying the protective effect of EEF1A1 against tumor development or progression. However, the tumor suppressive role of EEF1A1 in colon adenocarcinoma remains unclear. EEF1A1 expression has been determined to be independent of *KRAS* mutations in our study, suggesting that the expression of EEF1A1 is unlikely to involve *KRAS*-related colon adenocarcinoma. However, one study suggested that posttranslational changes in EEF1A1 might affect the KRAS pathway. Liu et al. suggested an association between EEF1A1 and *KRAS*-related tumorigenicity [29], demonstrating that neoplastic cell growth induced by oncogenic KRAS signaling may be associated with methylation of *EEF1A1* at the mRNA or protein level. Additionally, the results of the current study revealed that EEF1A1 expression was associated with tumor location, and right-sided colon cancers tended to show lower expression of EEF1A1, although the correlation with tumor sidedness was not significant. The biological role of EEF1A1 in colon adenocarcinoma and its correlation with tumor sidedness will be evaluated in our next study.

Based on our results, high expression of EEF1A1 indicates a favorable survival prognosis for patients with colon cancer, and at the mRNA level, *EEF1A1* was found to be downregulated in tumor tissues compared to that in normal tissues. Lin et al. revealed that EEF1A1 protein is overexpressed in estrogen/progesterone receptor (ER/PR)-positive and lymph node-negative breast cancers, which have relatively favorable outcomes compared to ER/PR-negative and lymph node-positive cases [30]. However, at the mRNA transcript level, low *EEF1A1* expression was an independent poor-prognostic factor with ER/PR-positive tumors; breast cancer-specific post-transcriptional regulatory events may be responsible for this discordance between the expression of *EEF1A1* mRNA and protein [30]. Further, Hassan et al. investigated the mRNA transcript levels of *EEF1A* isoforms across different cancers such as lung, breast, gastric, brain, prostate, liver, and colon cancers using the Oncomine and TCGA databases [31]. In colon cancer, *EEF1A1* mRNA expression levels were significantly low in patients with a high risk of recurrence compared to those in patients with a low risk, which was also consistent with our findings. Interestingly, based on our results, patients who did not undergo treatment for stage II colon cancer showed differences in OS according to EEF1A1 expression. In patients with stage II disease, EEF1A1 could serve as a potential biomarker for selecting candidates who may benefit from adjuvant treatment.

In contrast to our results, studies have reported that *EEF1A* downregulation leads to decreased expression of pAkt1, consequently inhibiting proliferation and invasion and promoting apoptosis in HCC (Hepato cellular carcinoma) cells [16]. In stomach cancer, EEF1A1 has been reported to promote gastric cancer cell migration and invasion [21]. Such contradictory reports regarding the role of EEF1A1 in tumorigenesis may be due to the fact that it plays fundamental roles in various cellular functions [12]. Even in the same cancer, EEF1A1 may act in an opposing manner with respect to different subtypes or stages. For example, Hassan et al. suggested that higher *EEF1A1* expression [31], which is otherwise a favorable biomarker for breast cancer, can be an indicator of poor OS, particularly in the basal subtype.

Our study has some limitations, such as its retrospective design. Further, the KRAS status was not verified in all populations. However, we analyzed a relatively homogeneous population, which compensates for these limitations. To the best of our knowledge, this is the first study to analyze the prognostic role of EEF1A1 in human colon cancer.

## 5. Conclusions

In conclusion, EEF1A1 may have a favorable prognostic effect on human colon adenocarcinoma, indicating that its loss can lead to poorer survival outcomes. Different expression levels of EEF1A protein in stages II and III of colon cancer may be independent prognostic markers of DFS and OS. Further studies are recommended to determine the mechanism underlying this correlation.

## Figures and Tables

**Table 1:** Baseline characteristics of colon cancer patients stratified based on EEF1A1 expression.

	EEF1A1-Low (*n* = 42)	EEF1A1-High (*n* = 239)	*p*-Value
Age (years)			
<65	22 (52.4%)	113 (47.3%)	0.616
≥65	20 (47.6%)	126 (52.7%)	
Sex			
Male	26 (61.9%)	129 (54.0%)	0.402
Female	16 (38.1%)	110 (46.0%)	
Body mass index (BMI) (kg/m^2)^			
<18.5	2 (4.8%)	17 (7.1%)	0.839
18.5–25.0	23 (54.7%)	124 (51.9%)	
>25.0	17 (40.5%)	98 (41.0%)	
Number of comorbid diseases			
0	23 (54.8%)	101 (42.3%)	0.265
1	10 (23.8%)	84 (35.1%)	
≥2	9 (21.4%)	54 (22.6%)	
Location of primary cancer			
Left	20 (47.6%)	152 (63.6%)	0.059
Right	22 (52.4%)	87 (36.4%)	0.059
Pre-operative carcinoembryonic antigen (CEA)			
<5 ng/mL	27 (64.3%)	151 (63.4%)	1.000
>5 ng/mL	15 (35.7%)	87 (36.6%)	
Bowel obstruction or perforation			
No	32 (76.2%)	215 (90.0%)	0.019
Yes	10 (23.8%)	24 (10.0%)	
Adjuvant chemotherapy			
No	11 (76.2%)	50 (20.9%)	0.424
Yes	31 (73.8%)	189 (79.1%)	
Stage (tumor/node/metastasis, TNM)			
II	15 (35.7%)	117 (49.0%)	0.132
III	27 (64.3%)	122 (51.0%)	
pT stage			
2	1 (2.4%)	10 (4.2%)	0.097
3	30 (71.4%)	197 (82.4%)	
4	11 (26.2%)	32 (13.4%)	
pN stage			0.076
0	15 (35.7%)	118 (49.4%)	
1	18 (42.9%)	62 (25.9%)	
2	9 (21.4%)	59 (24.7%)	
Differentiation			0.319
Well	1 (2.4%)	6 (2.5%)	
Moderate	36 (87.8%)	223 (93.3%)	
Poor	4 (9.8%)	10 (4.2%)	
Perineural invasion			0.083
No	21 (50.0%)	155 (64.9%)	
Yes	21 (50.0%)	84 (35.1%)	
Venous invasion			0.503
No	37 (88.1%)	199 (83.3%)	
Yes	5 (11.9%)	40 (16.7%)	
Lymphatic invasion			0.610
No	27 (64.3%)	141 (59.0%)	
Yes	15 (35.7%)	98 (41.0%)	
Number of harvested lymoh nodes	23.5 (10–63)	22 (4–115)	0.259
Number of metastatic lymph nodes	1 (0–15)	1 (0–18)	0.560
Metastatic lymphnodes/harvested lymph nodesratio	4.9 (0–66.7)	2.6 (0–100)	0.943
*KRAS* ^1^			0.811
Wild-type	14 (66.7%)	77 (63.1%)	
Mutant	7 (33.3%)	45 (36.9%)	

^1^*KRAS*; V-Ki-ras2 Kirsten rat sarcoma viral oncogene homolog gene.

**Table 2:** Cox regression analysis of overall survival for 281 patients with curatively-resected colon cancer.

	Crude Hazard Ratio (95% CI)	*p*-Value	Adjusted Hazard Ratio (95% CI)	*p*-Value
Age ≥ 65 (years)	5.08 (2.468–10.462)	<0.001	5.93 (2.810–12.493)	<0.001
Male	1.31 (0.738–2.316)	0.358		
BMI (reference: <18.5 kg/m^2^)				
18.5–25.0 (kg/m^2^)	1.17 (0.355–3.853)	0.796		
>25.0 (kg/m^2^)	0.98 (0.290–3.330)	0.978		
Number of comorbid diseases (reference: 0)				
1	1.02 (0.520–1.985)	0.964		
≥2	1.02 (0.776–2.963)	0.223		
Right-sided location of primary cancer	1.45 (0.827–2.527)	0.196		
Pre-operative CEA ≥ 5 ng/mL	1.794 (1.030–3.126)	0.039	1.76 (1.008–3.027)	0.058
Combined bowel obstruction or perforation at initial diagnosis	1.286 (0.578–2.859)	0.537		
Adjuvant chemotherapy	2.240 (1.247–4.024)	0.007		0.034
Stage III	1.509 (0.852–2.672)	0.158		
Differentiation (reference: well)				
Moderate	0.508 (0.123–2.096)	0.349		
Poor	0.531 (0.075–3.773)	0.527		
Perineural invasion	1.443 (0.827–2.516)	0.197		
Venous invasion	1.545 (0.791–3.017)	0.203		
Lymphatic invasion	1.542 (0.886–2.685)	0.120		
Number of harvested lymph nodes	0.996 (0.792–1.021)	0.756		
Number of metastatic lymph nodes	1.078 (1.004–1.156)	0.037	1.350 (0.718–2.539)	0.351
*KRAS*^1^ mutated cancer	2.020 (0.779–5.236)	0.148		
Low EEF1A1 expression	2.560 (1.380–4.749)	0.003	3.01 (1.636–5.543)	<0.001

^1^*KRAS*; V-Ki-ras2 Kirsten rat sarcoma viral oncogene homolog gene.

**Table 3:** Cox regression analysis of disease-free survival for 281 patients with curatively-resected colon cancer.

	Crude Hazard Ratio (95% CI)	*p*-Value	Adjusted Hazard Ratio (95% CI)	*p*-Value
Age ≥ 65 (years-old)	1.44 (0.852–2.442)	0.172		
Male	1.18 (0.697–2.008)	0.533		
BMI (reference: <18.5 kg/m^2^)		0.651		
18.5–25.0 (kg/m^2^)	0.69 (0.271–1.800)	0.457		
>25.0 (kg/m^2^)	0.98 (0.290–3.330)	0.978		
Number of comorbid diseases (reference: 0)				
1	1.04 (0.567–1.891)	0.909		
≥2	1.15 (0.593–2.216)	0.685		
Right-sided location of primary cancer	1.24 (0.730–2.103)	0.428		
Pre-operative CEA ≥ 5 ng/mL	2.35 (1.397–3.966)	0.001	2.23 (1.308–3.806)	0.003
Combined bowel obstruction or perforation at initial diagnosis	0.88 (0.379–2.057)	0.773		
Adjuvant chemotherapy	1.09 (0.578–2.066)	0.785		
Stage III	2.22 (1.256–3.908)	0.006	25.80 (3.087–215.562)	0.003
Differentiation (reference: well)		0.645		
Moderate	0.51 (0.123–2.096)	0.349		
Poor	0.53 (0.075–3.773)	0.527		
Perineural invasion	1.34 (0.796–2.268)	0.268		
Venous invasion	1.19 (0.604–2.364)	0.610		
Lymphatic invasion	1.52 (0.901–2.546)	0.117		
Number of harvested lymph nodes	1.01 (1.002–1.026)	0.021	1.02 (0.994–1.042)	0.150
Number of metastatic lymph nodes	1.10 (1.034–1.171)	0.003	0.24 (0.029–1.953)	0.182
KRAS ^1^ mutated cancer	1.42 (0.665–3.036)	0.364		
Low EEF1A1 expression	2.86 (1.619–5.042)	<0.001	2.54 (1.459–4.434)	<0.001

^1^*KRAS*; V-Ki-ras2 Kirsten rat sarcoma viral oncogene homolog gene.

## Supplementary Materials

The following are available online at https://www.mdpi.com/2077-0383/8/11/1903/s1, Table S1: Expression profile of EEF1A1.
