# Supplementary material for: Expression of EEF1A1 Is Associated with Prognosis of Patients with Colon Adenocarcinoma
Source: J Clin Med. 2019 Nov 7;8(11):1903. doi: 10.3390/jcm8111903 (PMC6912729; doi:10.3390/jcm8111903)
Supplement: Supplementary file 1 [file jcm-08-01903-s001.pdf]

Table S1. Expression profile of *EEF1A1*.

**Proportion score**		**Intensity score**		Composite score	
**0 (0-25%)**	**26 (9.3%)**	**0**	19 (6.8%)	0-2 (low)	42 (14.9%)
1 (26-50%)	26 (9.3%)	1	86 (30.6%)	3-6 (high)	239 (85.1%)
2 (51-75%)	40 (14.2%)	2	139 (49.5%)		
3 (76-100%)	189 (67.3%)	3	37 (13.2%)		
